# The role of transketolase in the immunotherapy and prognosis of hepatocellular carcinoma: a multi-omics approach

**DOI:** 10.3389/fimmu.2025.1529029

**Published:** 2025-03-31

**Authors:** Xuan-Yu Gu, Zheng-Jun Zhou, Hua Yao, Jia-Li Yang, Jin Gu, Rui Mu, Li-Jin Zhao

**Affiliations:** ^1^ Department of General Surgery, Digestive Disease Hospital, Affiliated Hospital of Zunyi Medical University, Zunyi, China; ^2^ Department of Critical Care Medicine, Affiliated Hospital of Zunyi Medical University, Zunyi, China

**Keywords:** transketolase, TKT, hepatocellular carcinoma, Hep-G2, pentose phosphate pathway

## Abstract

**Objective:**

To explore the role of transketolase (TKT) in the immunotherapy and prognosis of hepatocellular carcinoma (HCC).

**Materials and methods:**

TKT expression across various cancers and its associations with tumor immunity and prognosis were analyzed using nomogram models. A multi-omics approach was employed, including bulk RNA-seq analysis, methylation profiling, single-cell analysis, and spatial transcriptomics. Experimental methods included RT-qPCR, siRNA transfection, luciferase reporter assay, and chromatin immunoprecipitation.

**Results:**

TKT was significantly upregulated in multiple cancers and correlated with immune cell infiltration, particularly in HCC. Elevated TKT expression was associated with poor overall survival (OS) in HCC and was an independent prognostic factor (p < 0.05). Drug sensitivity analysis suggested that higher TKT expression was associated with reduced sensitivity to several chemotherapeutic agents, including sorafenib (p < 0.01). Furthermore, hypermethylation of the TKT promoter and low TKT expression were linked to improved OS in HCC (log-rank test p = 0.005). Single-cell analysis revealed that TKT was predominantly expressed in the monocyte/macrophage cluster associated with HCC, and pseudo-time series analysis highlighted TKT’s role in cell differentiation within this cluster. Spatial transcriptomics confirmed the close association between TKT and macrophage distribution in HCC. Moreover, STAT3 was found to directly regulate TKT expression by binding to its promoter region.

**Conclusion:**

Our findings suggest that TKT may play a role in tumor immunity and prognosis in HCC. Although these results provide insights into the potential involvement of TKT in immune cell infiltration and survival outcomes, further studies are required to fully elucidate its role in immunotherapy.

## Introduction

Hepatocellular carcinoma (HCC) is the sixth most common malignancy and the fourth leading cause of cancer-related mortality worldwide (1). While viral hepatitis and alcohol consumption remain the predominant causes of chronic liver disease globally, the increasing prevalence of obesity and metabolic syndrome in the United States has led to non-alcoholic fatty liver disease becoming one of the most common causes of chronic liver disease and HCC (2, 3). Curative treatments, such as surgical resection, organ transplantation, and ablation, are viable only for a subset of patients (4, 5). Therefore, it is imperative to elucidate the roles of key tumor molecular markers in the development and progression of HCC and to develop targeted therapies that specifically address these critical molecular drivers. In this context, multi-omics studies have gained prominence as powerful tools for identifying tumor molecular markers and key signaling pathways. By integrating multi-omics data, researchers can gain a more comprehensive and nuanced understanding of the molecular mechanisms underlying HCC development and progression, thereby providing new perspectives and a foundation for the development of innovative therapeutic strategies.

**Figure 1:**
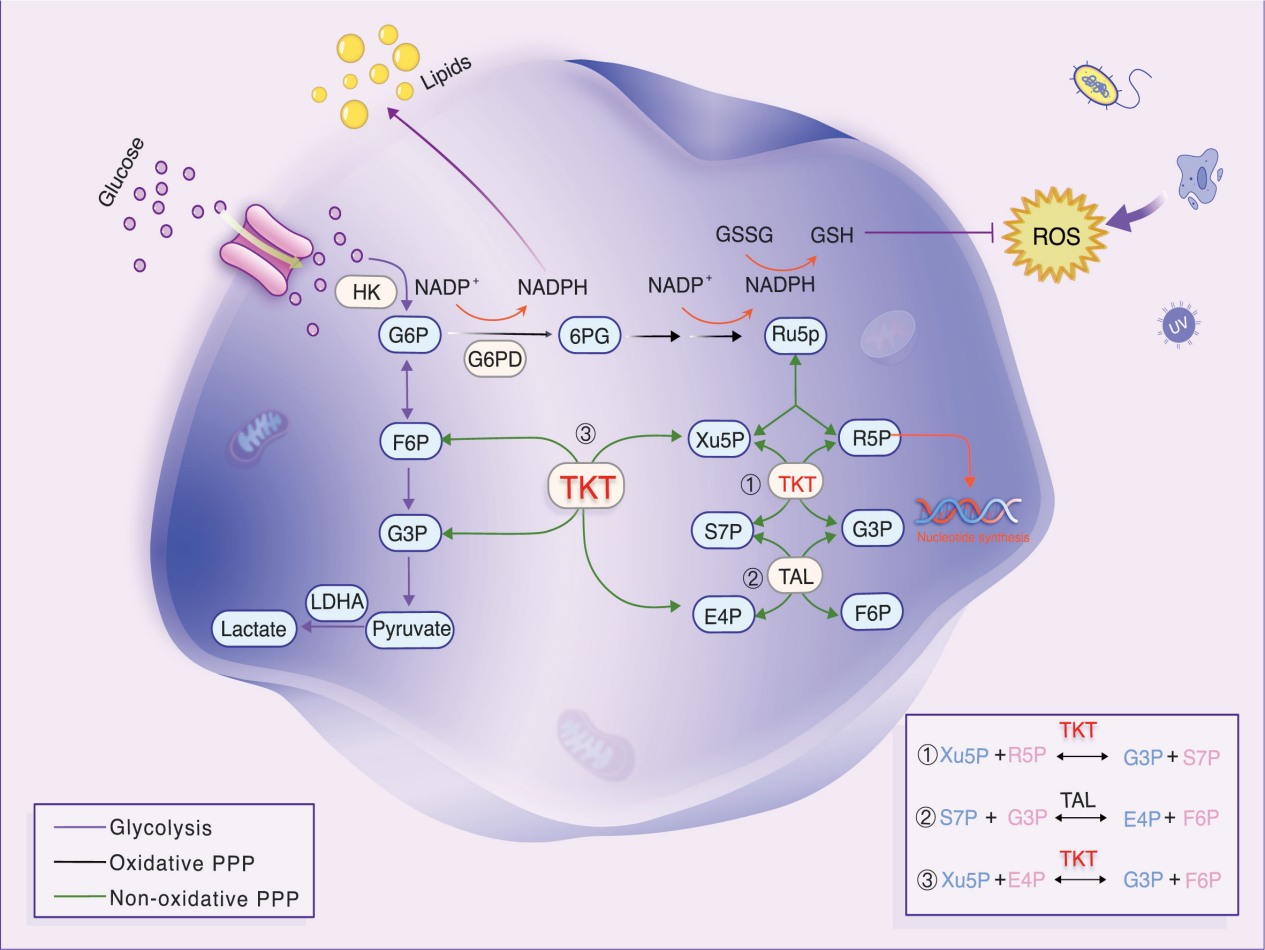
Diagram of glycolytic and PPP metabolism and transfer reactions in the non-oxidative PPP. TKT, a key enzyme, generates R5P. Reversible TKT reactions help the PPP adapt to metabolic needs. Under oxidative stress, TKT promotes F6P resynthesis, converting it to G6P, which boosts NADPH production and prevents oxidative stress.

In cancer cells, the Warburg effect manifests as elevated glycolysis through the pentose phosphate pathway (PPP), despite the presence of oxygen. Transketolase (TKT) enzyme reactions (6) regulate the non-oxidative phase of the PPP, producing more than 85% of ribose-5-phosphate (R5P), a crucial precursor for DNA and RNA biosynthesis (7). In the non-oxidative phase of the PPP, TKT mediates two key reversible reactions: the conversion of R5P and xylulose-5-phosphate (Xu5P) into glyceraldehyde-3-phosphate (G3P) and sedoheptulose-7-phosphate (S7P), and the transformation of Xu5P and erythrose-4-phosphate (E4P) into fructose-6-phosphate (F6P) and G3P (**Figure 1**) (8). The non-oxidative PPP is nearly universal across organisms, implying the widespread presence of TKT and its association with growth and development. The TKT-regulated pathway produces R5P and modulates NADPH levels. Both R5P and NADPH are critical for cell survival. NADPH, a major cellular antioxidant, reduces reactive oxygen species (ROS) levels and oxidative stress in cancer cells by sustaining the reduced state of glutathione (9). TKT is linked to resistance against chemoradiotherapy (10, 11). Targeted TKT inhibition reduces tumor growth and increases sensitivity to several chemotherapeutic drugs. Thus, TKT may be a new biomarker, and its inhibition could be a promising strategy for tumor treatment.

A correlation exists between the PPP and HCC, whereby the PPP is activated in HCC tissues, leading to an increase in the synthesis of TKT compared to non-tumor tissues (12). One study revealed that TKT enhances the expression of PD-L1 and VRK2 via the ROS-mTOR axis, thereby promoting immune evasion and HCC metastasis. Targeted or knocked-down expression of TKT resulted in a notable inhibition of FBXL6-induced immune evasion and HCC metastasis *in vitro* and *in vivo* (13). Moreover, an investigation of nine clinical HCC samples revealed a considerably elevated TKT expression in radiation-tolerant specimens relative to those exhibiting radiation sensitivity, indicating that TKT may be involved in radiation tolerance (10).

However, studies on the function of TKT in diverse tumors remain limited. In this study, we analyzed data from The Cancer Genome Atlas (TCGA) and Gene Expression Omnibus (GEO) databases, along with several clinical samples, to investigate the association between TKT expression and pathological features. Furthermore, we examined the correlation between TKT expression, tumor-infiltrating immune cells, tumor mutation burden (TMB), and pathway-related molecules. A prognostic nomogram was developed for patients with HCC to confirm TKT’s prognostic value in HCC. The link between TKT and immune cell infiltration was analyzed using bioinformatics, thus enhancing our understanding of the role of TKT in HCC prognosis and its potential in immunotherapy.

## Materials and methods

### Data source

RNA-seq data (in FPKM format) and clinicopathological information for 414 patients (363 HCC and 51 normal cases) were acquired from the TCGA database. The HCC dataset (GSE14520) was downloaded from the GEO database, containing 225 HCC and 220 normal cases. Additionally, the GSE97626 dataset contains data from three HCC cell lines with different metastatic potentials. Both TCGA and GEO databases were publicly available, with patient consent obtained. To ensure consistency, ENSEMBL Gene IDs were converted to Gene Symbol IDs, and genes expressed in less than 50% of samples were excluded.

### Pan-cancer analysis

The TCGAplot R package (14) was used to determine pan-cancer TKT levels. Pearson’s correlation analysis was employed to assess the relationship between TKT and established immunotherapy biomarkers, including immune cell infiltration, immune score, TMB, and other well-known immune checkpoint (ICP) genes. The cBioPortal tool (http://www.cbioportal.org/) was used to detect TKT mutations in cancers. Univariate Cox regression and the Kaplan–Meier (K-M) method were used to illustrate TKT’s prognostic role. Continuous TKT expression variables were utilized in the univariate Cox regression, while high- and low- TKT expression was utilized in the K-M curve analysis, with the cutoff chosen by the “surv-cutpoint” function of the “survminer” R package.

### Identification of differentially expressed genes between low- and high-TKT expression subgroups

The samples were divided into the high- and low-expression groups based on the median TKT expression level. DEGs between the two groups were identified using the R package ‘Limma,’ with significance criteria defined as an adjusted P-value < 0.05 and |log2- FC| > 1. The DEGs were visualized through volcano plots, which were created using the “ggplot2” R package.

### Nomogram establishment

Univariate and multivariate Cox regression models were applied to investigate the impact of clinical factors (age, sex, grade, stage, and TNM.T) and TKT expression on overall survival (OS) in patients with HCC. Receiver-operating characteristic (ROC) curves were plotted to determine the prognostic power of the nomograms. Decision curve analysis (DCA) and calibration curves validated the agreement between observed survival outcomes and those predicted by the nomograms.

### Methylation, protein expression, and transcription factor analysis methods

The correlation between methylation and mRNA levels of the signature genes was evaluated using the “corrplot” package, while the relationship between DNA methylation and OS was assessed by K-M survival analysis. Methylation data were obtained from the Illumina Human Methylation 450K platform, including detailed annotations, such as IllmnID, UCSC RefGene Names, and UCSC RefGene Group. TKT protein expression data from the HCC cohort were retrieved from the TCPA and Clinical Proteomic Tumor Analysis Consortium (CPTAC) databases to complement these analyses, providing a more comprehensive understanding of TKT regulation at both transcriptional and protein levels. To further investigate the transcriptional regulation of TKT, five web tools—hTFTarget, ENCODE, ChIP_Atlas, GTRD, and KnockTF—were used to identify potential transcription factors (TFs). Additionally, TF-binding sites were predicted using the JASPAR database (https://jaspar.elixir.no/), providing insights into the regulatory mechanisms underlying TKT expression.

### Single-cell and spatial transcriptome analysis of TKT

Single-cell RNA sequencing (scRNA-seq) data from GSE140228 were downloaded from the TISCH database and processed using Seurat V4 (15). After applying stringent quality control (UMI > 200, mitochondrial gene content < 20%, and log10 genes per nUMI > 0.8), the filtered cells were visualized using UMAP. These cells were manually annotated with CellMarker 2.0, and cell type markers were identified through the “FindAllMarkers” function (min. pct = 0.25, logfc.threshold = 1, test.use = “wilcox”). Monocle 2 was applied to further explore the expression patterns of TKT and immune activation markers in the immune pseudotime trajectories of HCC, reducing dimensionality with DDRTree, and visualizing gene expression trends.

Spatial transcriptome (ST) data from HCC samples (GSE203612, GSE238264) were incorporated to investigate the spatial architecture of the tumor microenvironment. By integrating ST data with single-cell transcriptomics, we performed reverse convolution analysis to deconvolute cellular composition on 10xVisium slides. A comprehensive scRNA-seq reference library was established from the same cancer type using quality control measures to ensure the reliability of the scRNA-seq data. A signature score matrix was developed by averaging the top 25 genes in each cell type in the scRNA-seq reference. This matrix was used to generate an enrichment scoring matrix using the Cottrazm package, which facilitated cellular composition analysis. SpatialFeaturePlot in Seurat was used to visualize these enrichment scores, with higher scores corresponding to a greater abundance of specific cell types in each spot.

### Colocalization

Data for bbj-a-158 and eqtl-a-ENSG00000163931 were obtained from the IEU OpenGWAS project. We utilized the “coloc” package for colocalization analysis between TKT’s eQTL and HCC, applying default priors (P1 = 10^-4^, P2 = 10^-4^, P12 = 10^-5^). These parameters estimate the likelihood that an SNP is relevant to gene expression, HCC, or both. The analysis generated the posterior probabilities for five potential relationships: no association (H0), association with only one trait (H1 or H2), two separate association signals (H3), and a shared association signal (H4). The H4 model, indicating a common causal variant, was preferred, and results with a PP4 value greater than 0.75 were considered significant for dual associations (16).

### Experimental validation of TKT expression and regulation

The HepG2, THLE2, and 293T cell lines were cultured in specific media purchased from Pricella (Wuhan, China) under conditions of 37°C and 5% CO2. HepG2 cells were applied to model HCC, THLE2 cells served as normal liver cell controls, while 293T cells were used for dual-luciferase reporter assays to investigate the transcriptional regulation of TKT. Three liver cancer (LIHC) and normal tissue samples were obtained from the Affiliated Hospital of Zunyi Medical University to investigate TKT expression. Total RNA from the cells and tissues was extracted with a TRIzol RNA extraction kit and reverse-transcribed into cDNA using a reverse transcription kit. The expression levels of TKT were quantified using RT-qPCR, while gene suppression effects were calculated using the 2-ΔΔCt method. Detailed primer sequences are provided in **Supplementary Table S1**.

Subsequently, HepG2 cells were reverse-transfected with siRNA using RNAiMAX, and further experiments were conducted 24 h post-transfection. Among the three candidate siRNAs, si-2 and si-3 were selected for further study; siRNA sequences are detailed in **Supplementary Table S2**. Cell viability was assessed using the CCK8 assay, with cells seeded at a density of 1.0 × 10^4^ cells/well in 96-well plates. After 2 h of adhesion, 10 µl of CCK-8 solution was added at designated time points (0, 12, 24, 36, and 48 h) and incubated at 37°C in a 5% CO2 humidified environment for 1 h, followed by absorbance measurement at 450 nm using a spectrophotometer.

Additionally, protein concentration, glucose, and lactate levels were measured using the Enhanced Bradford Protein Assay Kit, O-Toluidine Glucose Assay Kit, and L-Lactic Acid Colorimetric Assay Kit, respectively. The experiments were conducted in strict accordance with the manufacturer’s instructions. Protein concentrations were adjusted using the BCA assay.

To explore the transcriptional regulation of TKT, 293T cells were co-transfected with either the empty plasmid pGL3-TKT-wt or pGL3-TKT-mut, along with pcDNA-3.1 or pcDNA-3.1-STAT3 mimics, using Lipofectamine 2000. After 48 h, luciferase activity was measured using a Dual-Luciferase Reporter Gene Detection System. ChIP assays were performed to verify the binding of STAT3 to the TKT promoter regions. Immunoprecipitation was conducted using an anti-STAT3 antibody or IgG control, and the relative enrichment of the target gene was quantified using RT-qPCR. Primer sequences are provided in **Supplementary Table S1**.

### Statistical analysis

The prognostic power of TKT in various cancers was evaluated using univariate Cox regression analysis and the K-M method, while Pearson’s correlation was applied to explore the relationships between TKT and other factors. DEGs were mapped to gene symbols using the “org.Hs.eg.db” (v3.10.0) R package, followed by functional annotation and gene set variation analysis (GSEA) using the “ClusterProfiler” (v3.14.3) R package with thresholds set at P < 0.05, FDR < 0.25, and |NES| > 1 for significant enrichment of items and pathways (17, 18). Gene set variation analysis (GSVA), a non-parametric and unsupervised method for assessing gene set enrichment in expression data, was performed to investigate hallmark function differences between high and low TKT expression groups using reference gene sets from the MsigDB file (h.all.v2023.2.Hs.symbols.gmt) (19). Chemotherapy drug response prediction was also conducted using a similar approach. All *in vitro* experimental data are presented as means ± standard deviation and analyzed using Prism 10 software, with P < 0.05 indicating statistical significance.

## Results

### TKT expression and its prognostic value in pan-cancers

**Figure 2:**
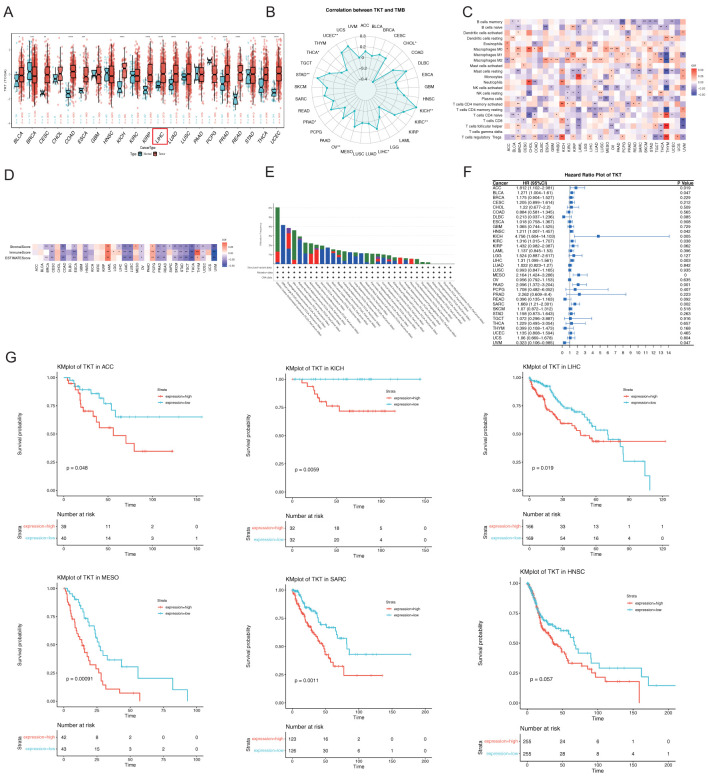
Comprehensive analysis of TKT gene expression, immune infiltration analysis, and their relationship with prognosis in pan-cancer. **(A)** TKT levels in tumors and adjacent normal tissues from TCGA. **(B)** Relationship between TKT levels and TMB in TCGA. **(C)** Correlation between TKT levels and immune cell ratios. **(D)** Relationship between TKT levels and immune scores. **(E)** Frequency and types of genetic alterations in TKT. **(F)** Cox regression analysis of TKT expression across TCGA cancers. **(G)** K-M plots showing survival analysis of TKT levels in ACC, KICH, LIHC, MESO, SARC, and HNSC. (*P <0.05, **P < 0.01, ***P < 0.001, ****P < 0.0001).

RNA-seq expression data from the TCGA demonstrated that TKT was significantly overexpressed in several cancers, including bladder cancer (BLCA), cervical cancer, colon cancer, esophageal cancer, kidney chromophobe (KICH), kidney papillary cell carcinoma, LIHC, lung adenocarcinoma, prostate cancer (PRAD), rectal cancer, thyroid cancer (THCA) and uterine corpus endometrial cancer (UCEC), but exhibited low expression levels in breast cancer (**Figure 2A**). After further typing of the TCGA breast cancer data, we categorized breast cancer cases into triple-negative breast cancer (TNBC) and non-triple-negative breast cancer (non-TNBC) (**Supplementary Figure S1A**). The analysis showed that the expression of TKT was significantly higher in TNBC than non-TNBC cases. This phenomenon may be related to the differences in metabolic alterations among tumor subtypes. However, it is worth noting that non-TNBC occupied a larger proportion of the dataset, which may have affected the interpretation of the overall analysis results. Non-TNBC is usually accompanied by different metabolic profiles, which may have masked the expression of specific metabolic markers for TNBC subtypes in some cases (20). **Supplementary Figure S1B** presents the physiological expression profiles of TKT in different tissues of the human body from the GTEx database.

Moreover, TKT expression was found to be negatively correlated with TMB in CHOL, ovarian cancer, and THCA, but positively correlated in KICH, KIRC, LIHC, PRAD, STAD, and UCEC (**Figure 2B**). Additionally, we found a positive association between TKT and macrophages in most cancers and a negative correlation with some effector immune cells, such as resting CD4+ memory T cells and activated natural killer (NK) cells (**Figure 2C**). This immune modulation may support an immunosuppressive milieu, promoting tumor growth and immune evasion. The negative correlation of TKT expression with immune and stromal scores (**Figure 2D**) highlights its role in influencing the density and activity of non-tumor cells within the tumor stroma, further suggesting that TKT expression may be linked to a less active immune environment. Analysis of TCGA cohorts revealed frequent TKT alterations, particularly in UCEC (6.05%) (**Figure 2E**), which may underlie the specific phenotypic traits of this cancer type. Overall, these findings suggest that TKT may play a role in cancer cell metabolism and immune interactions, making it a promising target for further investigation in precision oncology strategies.

The Cox proportional hazards model revealed significant associations between TKT expression and OS across multiple cancer types, including mesothelioma (MESO) (p < 0.001), pancreatic cancer (P = 0.001), sarcoma (SARC) (P = 0.002), LIHC (P = 0.003), KICH (P = 0.005), adrenocortical cancer (ACC) (P = 0.019), KIRC (P = 0.038), HNSC (P = 0.042), BLCA (P = 0.047), and ocular melanoma (UVM) (P = 0.047) (**Figure 2F**). TKT was identified as a high-risk factor in most cancers, particularly KICH, where it exhibited the highest HR (4.756), suggesting that elevated TKT expression dramatically increased the risk of mortality in these patients. Conversely, TKT is a low-risk parameter in UVM, indicating a protective role or less aggressive tumor phenotype associated with its expression in this context. K-M survival analysis corroborated these findings, showing that elevated TKT levels were associated with significantly shorter OS in ACC, KICH, LIHC, MESO, SARC, and HNSC (**Figure 2G**). These results highlight the prognostic potential of TKT, as its expression not only influences tumor biology but also serves as a critical determinant of patient outcomes. The strong correlation between TKT expression and poor survival in various cancers suggests its potential prognostic value, warranting further investigation into its role in informing therapeutic decisions.

### Expression and prognostic value of TKT in HCC

**Figure 3:**
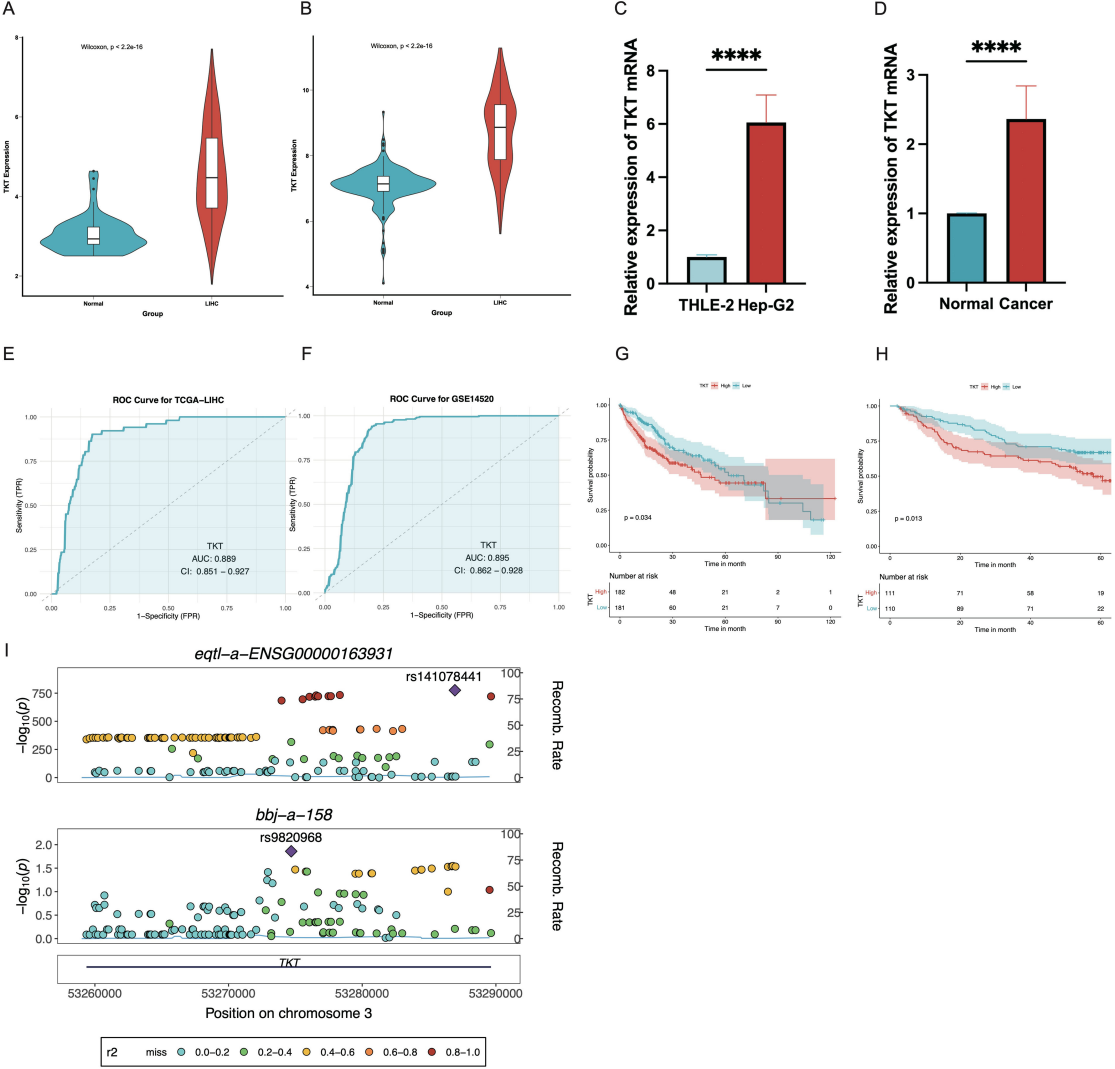
Expression levels and prognostic value of TKT in the TCGA dataset and GEO database were analyzed. **(A)** TKT levels were elevated in HCC tissues in the TCGA data. **(B)** Similar results were observed using GSE14520 data, showing higher TKT expression in HCC tissues. **(C, D)** RT-qPCR confirmed increased TKT expression in tumor cells, when using β-actin as the normalization control (****P ≤ 0.0001). **(E, F)** ROC curve analysis for TKT expression in TCGA-LIHC and GSE14520 datasets, with the x-axis denoting the false positive rate and the y-axis the true positive rate. AUC values were categorized as low (0.5–0.7), medium (0.7–0.9), and high (0.9–1.0) predictive effects. CI indicates confidence interval. **(G, H)** K-M survival curves assessed the prognostic significance of TKT expression in the TCGA-LIHC and GSE14520 cohorts. **(I)** Regional Manhattan plot of associations of TKT and HCC risk. The lead SNP is displayed as a purple diamond.

TKT mRNA levels were notably elevated in HCC tissues compared with normal liver tissues (**Figure 3A**), consistent with the GEO database data (GSE14520) (**Figure 3B**). RT-qPCR analysis of matched HCC and normal liver samples further validated the increased TKT mRNA levels in HCC tissues (**Figures 3C, D**). ROC curve analysis revealed a high diagnostic accuracy for TKT in distinguishing HCC from normal liver tissues, with AUC values of 0.889 (CI = 0.851–0.927) for TCGA-LIHC and 0.895 (CI = 0.862–0.928) for GSE14520 (**Figures 3E, F**). This indicates that TKT may be a reliable diagnostic biomarker for HCC. Furthermore, we examined the influence of TKT levels on OS in patients with HCC by categorizing them into high and low TKT groups according to the median level. K-M analysis revealed that patients with higher TKT levels had markedly poorer OS in both the TCGA-LIHC and GSE14520 cohorts (**Figures 3G, H**, **Table 1**). A colocalization analysis was conducted to explore potential genetic links between TKT and HCC susceptibility, revealing a strong association between TKT expression and HCC risk loci (PP4 = 1.00) (**Figure 3I**, **Supplementary Table S3**). This genetic colocalization suggests that TKT may be crucial in HCC pathogenesis, reinforcing its value as a diagnostic and prognostic biomarker. These findings provide substantial evidence for the role of TKT in HCC development and progression, suggesting its potential utility as a diagnostic and prognostic biomarker. Further studies are needed for clinical applications.

**Table 1:** TCGA-LIHC and GSE14520 clinical baseline table.

Datasets	Variable	Level	Alive	Dead	P
	n		233	130	
TCGA-LIHC	Gender	female	68 (29.2)	50 (38.5)	0.091
		male	165 (70.8)	80 (61.5)	
	Age	<60	118 (50.6)	55 (42.3)	0.157
		>60	115 (49.4)	75 (57.7)	
	Grade	G1-G2	152 (65.5)	78 (61.9)	0.572
		G3-G4	80 (34.5)	48 (38.1)	
	Stage	Stage I-II	185 (83.0)	69 (59.5)	<0.001
		Stage III-IV	38 (17.0)	47 (40.5)	
	TNM.T	T1-T2	194 (84.0)	77 (59.7)	<0.001
		T3-T4	37 (16.0)	52 (40.3)	
	n		146	96	
GSE14520	Gender	Female	23 (15.8)	8 (8.3)	0.135
		Male	123 (84.2)	88 (91.7)	
	Age	<60	118 (80.8)	78 (81.2)	1.000
		>60	28 (19.2)	18 (18.8)	
	TNM staging	I	75 (54.0)	21 (24.4)	<0.001
		II	46 (33.1)	32 (37.2)	
		III	18 (12.9)	33 (38.4)	

### Clinical application of a nomogram incorporating the expression of TKT

**Figure 4:**
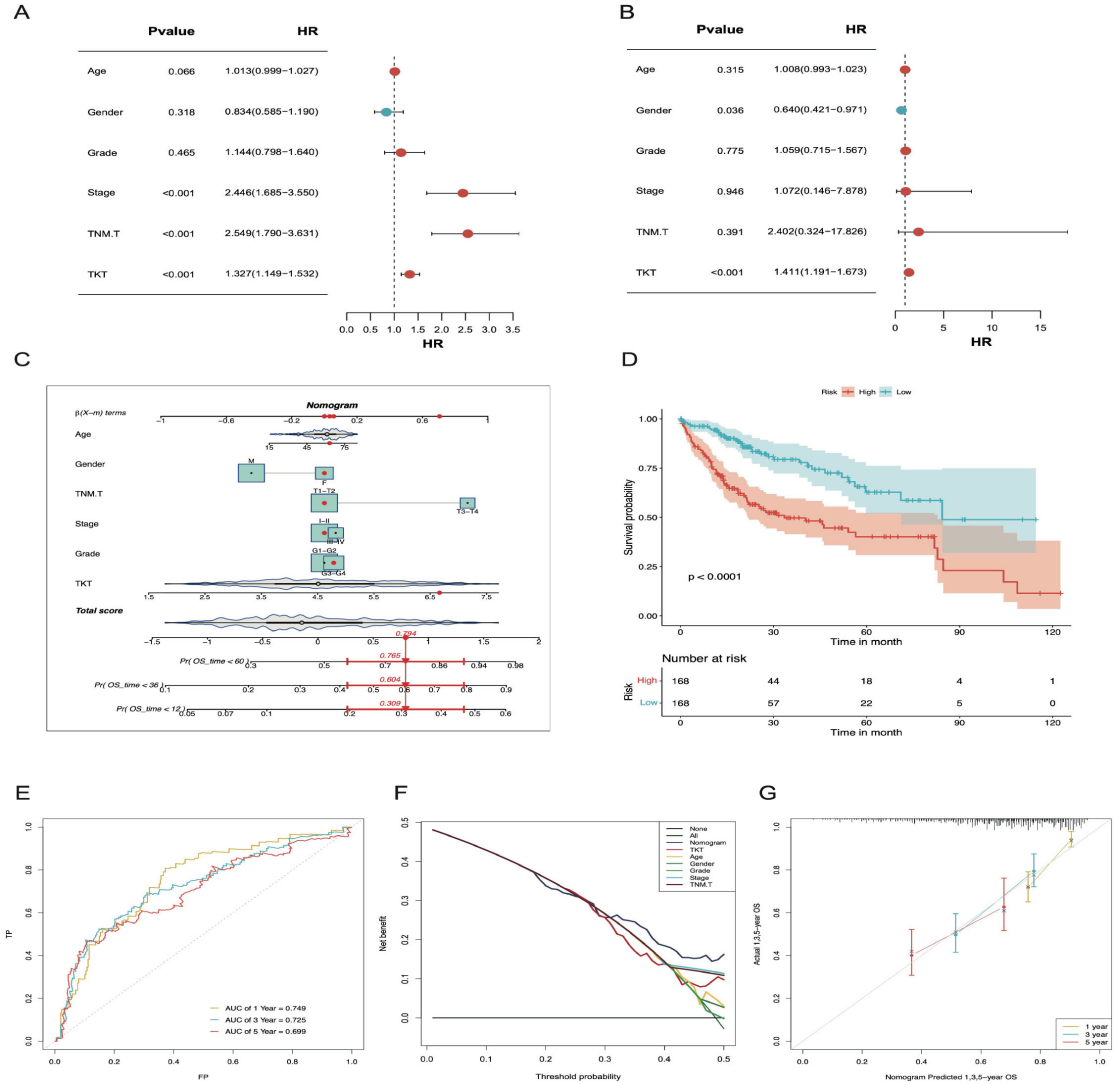
The nomogram constructed and validated based on TKT expression. **(A)** Univariate Cox regression forest plot showing the link between TKT mRNA level and OS in HCC patients with various clinicopathological features. **(B)** Multivariate Cox regression forest plot illustrates the link of TKT mRNA expression with OS in HCC patients, considering multiple clinicopathological factors. **(C)** A nomogram was constructed using risk scores and clinical features to predict OS in HCC patients. **(D)** K-M survival curves for HCC patients, stratified by the nomogram, indicate remarkably better OS for the low-risk group (p < 0.0001). **(E)** The AUC values for forecasting 1-, 3-, and 5-year OS were 0.749, 0.725, and 0.699, respectively. **(F)** DCA for the nomogram. **(G)** Calibration plots show that the observed OS of HCC patients aligns closely with the nomogram-predicted OS.

Cox analyses indicated that TKT expression was an independent prognostic factor in HCC (**Figures 4A, B**). To enhance its clinical applicability, we developed a comprehensive nomogram that integrated TKT expression with other clinical features to predict OS in patients with HCC based on the TCGA data (**Figure 4C**). This integrated model demonstrated a significantly improved predictive performance over TKT expression alone, as evidenced by a clear distinction in OS between the low- and high-risk groups (P < 0.0001; **Figure 4D**). The model’s predictive accuracy was further validated using ROC curve analysis, with AUC values of 0.749, 0.725, and 0.699 for 1-, 3-, and 5-year OS predictions, respectively (**Figure 4E**), demonstrating its robust prognostic capability. These AUC values indicate a stable and reliable model performance across different time points, highlighting the enhanced precision achieved by integrating multiple parameters. DCA further reinforced the clinical benefit of this nomogram, showing a higher net benefit across a range of threshold probabilities compared to using TKT or other clinical features alone (**Figure 4F**). Additionally, calibration curves demonstrated strong agreement between the predicted and observed OS at 1, 3, and 5 years (**Figure 4G**), confirming the nomogram’s predictive consistency and reliability. Overall, the nomogram effectively integrated TKT expression with other clinical parameters, offering a powerful tool for risk stratification and personalized prognosis assessment in patients with HCC.

### TKT involvement in key signaling pathways

**Figure 5:**
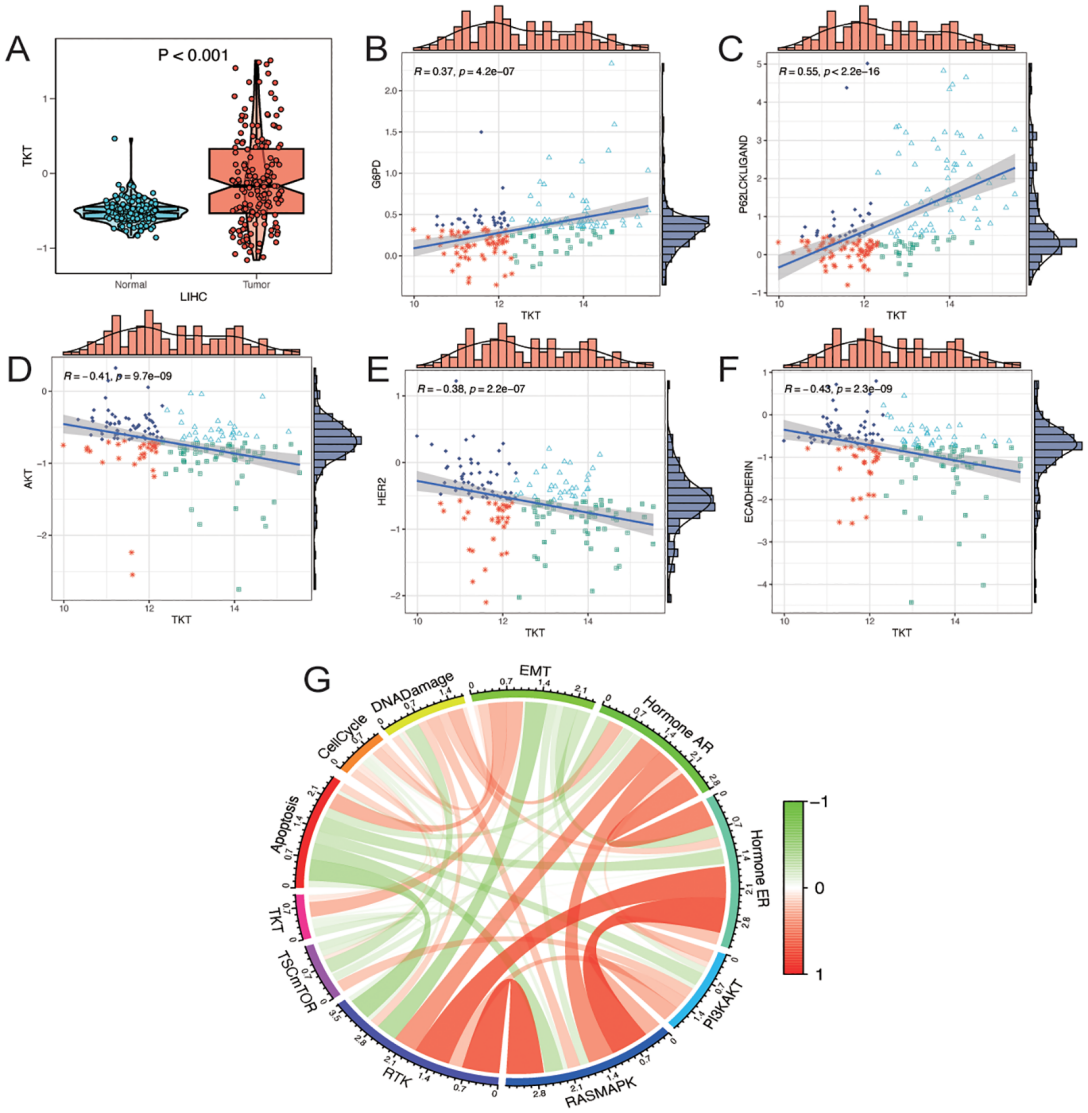
Analysis of TKT proteomics. **(A)** Differences in TKT total protein levels (mass spectrometry) in the CPTAC database between tumor and normal groups. **(B–F)** Correlation analysis between TKT expression and functional protein levels by TCPA-RPPA sequencing. **(G)** Correlation of gene expression of TKT with pathway levels quantified by TCPA-RPPA sequencing of functional proteins.

In the CPTAC-LIHC cohort, TKT protein levels were significantly elevated in tumor tissues compared to normal tissues (**Figure 5A**), suggesting a potential role in the pathogenesis of LIHC. Analysis of the CPTAC-LIHC dataset indicated a positive correlation between TKT proteomic expression and the pentose phosphate and p62 pathways (**Figures 5B, C**). These correlations imply that TKT may be involved in various metabolic processes critical for tumor progression, such as nucleotide biosynthesis and redox balance, which are essential for cancer cell survival and proliferation. Additionally, its association with the p62 pathway suggests a potential role in modulating autophagy-related processes, although further investigation is needed to determine whether these pathways are directly regulated by TKT or whether TKT is a downstream effector of shared upstream signals. In contrast, TKT expression was negatively correlated with the Akt, HER2, and E-cadherin pathways (**Figures 5D–F**), suggesting that while TKT may be associated with these pathways, it is more likely that this relationship arises from shared regulatory mechanisms rather than TKT directly influencing these pathways.

To further investigate the involvement of TKT in cancer biology, we assessed its relationship with 10 cancer-related pathways—TSC/mTOR, RTK, PI3K/AKT, RAS/MAPK, hormone ER, hormone AR, DNA damage response, cell cycle, epithelial–mesenchymal transition (EMT), and apoptosis—using protein expression data from the RPPA in the TCPA database (21). TKT expression was positively associated with the EMT pathway, which is crucial for tumor metastasis and invasion (**Figure 5G**). This correlation with EMT was accompanied by a negative correlation with E-cadherin, a key epithelial marker that is typically downregulated during EMT. These findings suggest that TKT may promote EMT by affecting E-cadherin expression, but the mechanism remains unclear. Overall, these findings highlight TKT as a potential modulator of multiple oncogenic pathways that may contribute to tumor growth and metastasis in HCC. However, further functional studies are required to establish causality.

### TKT methylation status

**Figure 6:**
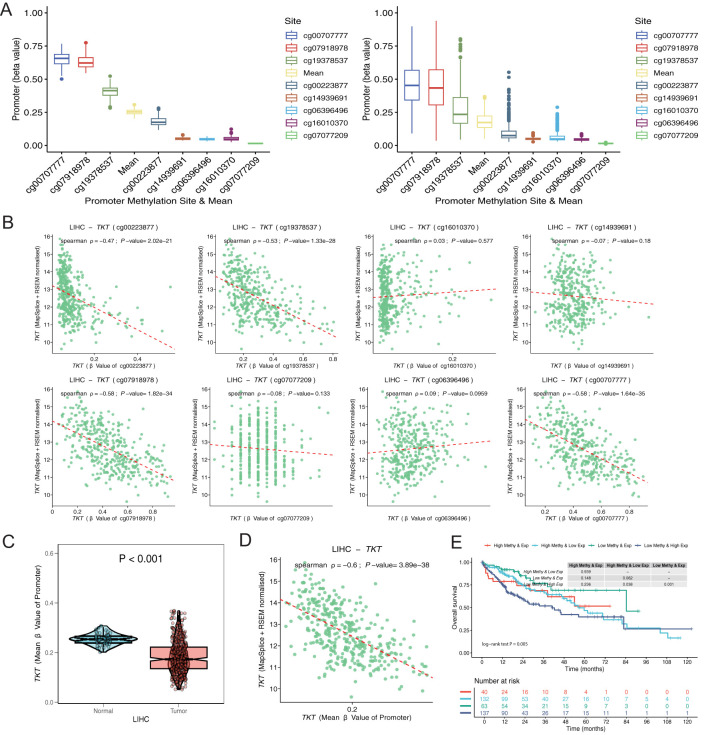
Exploration of TKT promoter cg sites. **(A)** Distribution of methylation levels for each and all sites averaged over normal and tumor tissues. **(B)** Correlation of each promoter site with gene expression in tumor tissues. **(C)** Differences in methylation levels between normal and tumor tissues for the mean values of all sites. **(D)** Correlation of mean values of all sites with gene expression in tumor tissue. **(E)** K-M survival analysis of 4 survival periods for genetic molecular subtypes.

**Figures 6A–D** illustrates the inverse relationship between TKT expression and methylation levels across promoter CpG sites, each displaying distinct beta values representing their correlation coefficients with TKT expression. Based on TKT promoter methylation and mRNA expression levels, we categorized patients into four groups: high methylation and high expression, low methylation and high expression, high methylation and low expression, and low methylation and low expression. The subgroup with high methylation and low TKT expression in the promoter region exhibited the most favorable OS, suggesting a potential protective effect of promoter hypermethylation. In contrast, the subgroup characterized by low methylation and high TKT expression had the poorest OS, indicating a potentially unfavorable prognosis (**Figure 6E**). These findings suggest that hypermethylation of the TKT promoter suppresses its expression, which could confer a survival advantage to patients with HCC by limiting TKT-driven tumor progression. Conversely, the hypomethylation-associated upregulation of TKT expression may enhance its oncogenic potential, leading to worse patient outcomes. These findings indicate that the TKT promoter methylation status might be useful in predicting patient prognosis and could offer a promising avenue for developing targeted therapies for HCC, pending further validation.

### TKT expression correlates with tumor immune microenvironment in HCC

**Figure 7:**
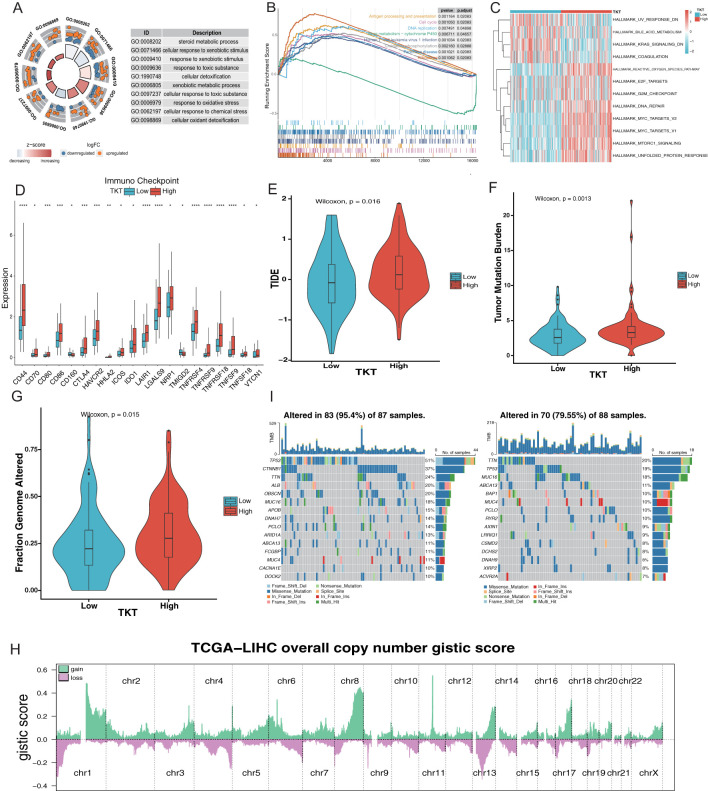
Relationship between TKT expression and the microenvironment of HCC. **(A)** Enrichment analysis of GO terms. **(B)** Enrichment analysis of KEGG pathways associated with TKT expression. **(C)** GSVA enrichment for TKT visualized using a heatmap. **(D)** TKT shows significant correlations with ICPs across various cancers. **(E)** Prediction of immunotherapy response in HCC patients based on TKT expression using the TIDE algorithm. **(F)** Relationship between TKT expression and TMB. **(G)** Relationship between TKT expression and FGA. **(H)** Distribution of Overall CNV GISTIC Score in TCGA-LIHC. **(I)** Comparison of mutation profiles between HCC patients with high and low TKT expression. (*P <0.05, **P < 0.01, ***P < 0.001, ****P < 0.0001).

Gene ontology (GO), Kyoto Encyclopedia of Genes and Genomes (KEGG), GSEA, and GSVA analyses revealed that the DEGs related to TKT were associated with several critical GO biological processes, including steroid metabolic processes, cellular responses to xenobiotic stimuli, xenobiotic metabolic processes, and responses to oxidative stress (**Figure 7A**). These associations suggest that TKT may be central in metabolic reprogramming in HCC, particularly in processes related to detoxification, hormone metabolism, and oxidative stress response—key pathways that cancer cells exploit to support survival under adverse conditions. The ability of TKT to modulate these processes underscores its potential function in enhancing cancer cell resistance to external stressors such as chemotherapy or oxidative damage, contributing to tumor persistence.

GSEA analysis of the KEGG pathways revealed that the high-TKT group was enriched in pathways involved in antigen processing and presentation, DNA replication, cell cycle, Human T-cell leukemia virus 1 infection, oxidative phosphorylation, and ribosome biogenesis (**Figure 7B**). The enrichment of pathways related to DNA replication and the cell cycle suggests that TKT expression may drive increased proliferative activity in tumor cells, promoting uncontrolled cell division—a hallmark of cancer. Moreover, the involvement of oxidative phosphorylation and ribosomal activity indicates that TKT may enhance the metabolic capacity of tumor cells, enhancing energy production and protein synthesis needed for tumor growth.

In contrast, GSVA analysis demonstrated that UV response downregulation, bile acid metabolism, KRAS signaling downregulation, and coagulation pathways were enriched in the low TKT group, highlighting reduced metabolic and stress response activity in these cells. In contrast, high TKT expression positively correlated with E2F targets, ROS pathway, DNA repair, and MYC targets (v1 and v2) (**Figure 7C**). The activation of the ROS pathway in the high TKT group suggests that TKT may help cancer cells manage oxidative stress, an important survival mechanism in hypoxic tumor environments. The positive association with E2F and MYC targets further implies that TKT supports tumor proliferation and progression by regulating TFs involved in cell cycle progression and metabolic control. These findings indicate that high TKT expression promotes the metabolic and proliferative aggressiveness of HCC cells.

A comprehensive analysis of ICPs revealed significantly higher levels of ICPs such as CD44, CD80, CD86, and CTLA-4 in the high-TKT expression group (**Figure 7D**), thereby allowing tumor cells to evade immune surveillance, and facilitating tumor progression (**Supplementary Figure S1C**). The correlation between TKT and HLA gene expression (**Supplementary Figure S1D**) further highlights its involvement in immune modulation, influencing tumor-immune system interactions. The lower TIDE score observed in the low TKT group (**Figure 7E**) suggests that tumors with low TKT expression may be more responsive to immunotherapy, while high TKT expression could predict poorer immunotherapy outcomes due to enhanced immune suppression.

Further analysis revealed a relationship between TKT and hypoxia-responsive gene expression, with a higher hypoxia score in the high TKT group (**Supplementary Figure S1E**). This association highlights its role in supporting tumor adaptation to hypoxic microenvironments, a common feature in aggressive cancers. Hypoxia induces metabolic shifts, promotes angiogenesis, and enhances tumor survival, while TKT may be intricately involved in these adaptive processes. To further explore the relationship between TKT expression and HCC metastaticity, we subsequently analyzed the expression levels of TKT in several HCC cell lines with different metastatic potentials (Huh7, MHCC97L, and HCCLM3) in the GSE97626 dataset. The results showed that TKT expression in the high metastatic cell line HCCLM3 was significantly higher than that in the low metastatic cell line Huh7, while MHCC97L cells showed intermediate levels (**Supplementary Figure S1F**). Based on these results, we hypothesized that TKT may play an important role in promoting HCC metastasis. Further, high enrichment scores for stemness gene sets in the high TKT group (**Supplementary Figure S1G**) suggest that TKT may be involved in maintaining cancer stem cell properties such as unlimited proliferation and self-renewal, contributing to tumor heterogeneity, recurrence, and resistance to therapies.

Genomic analysis revealed a positive correlation between high TKT levels and increased TMB, as well as a higher fraction of FGA (**Figures 7F, G**). This association between TKT and genomic instability suggests that TKT may promote mutagenic processes, driving genetic diversity within tumors, which could lead to the emergence of more aggressive or therapy-resistant clones that contribute to poor prognosis. The significant chromosomal alterations identified through CNV analysis (**Figure 7H**) in the high-TKT expression group, along with the higher mutation frequencies of key driver genes such as TP53 and CTNNB1 (51% vs. 19% and 37% vs. 7%, respectively) (**Figure 7I**).

Overall, these findings suggest that TKT plays multiple roles in HCC by influencing metabolic reprogramming, cell proliferation, immune evasion, and genomic instability. The involvement of TKT in oncogenic pathways and its effects on the tumor microenvironment highlight its potential as a therapeutic target. However, further studies are needed to explore its suitability for combination therapies aimed at inhibiting metabolic pathways, enhancing immune responses, or addressing genomic instability.

### ScRNA-Seq and spatial transcriptome analysis of HCC

**Figure 8:**
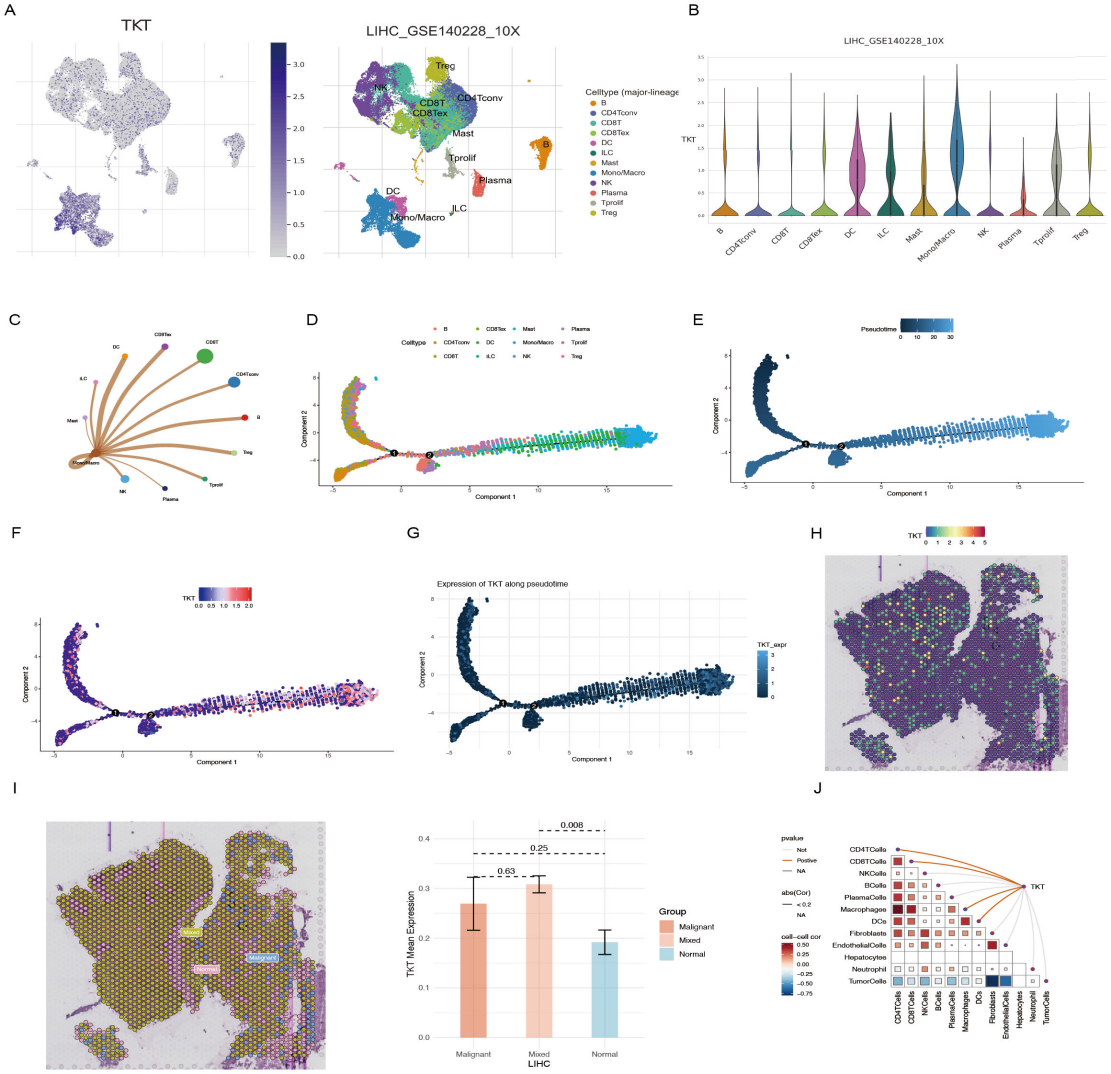
scRNA-Seq and spatial transcriptome analysis of TKT in HCC. **(A, B)** The relationship between TKT expression levels and various immune cell clusters. **(C)** Cell-cell interactions between mono/macro and other immune cell clusters. **(D)** Cell type trajectory. **(E)** General cell trajectory. **(F)** TKT in Mono/Macro trajectory. **(G)** TKT pseudotime trajectory. **(H)** ST localization of TKT. **(I)** Differential expression of TKT in malignant, mixed malignant, and normal regions. **(J)** Spearman correlation of TKT expression with microenvironmental components at ST resolution.

Overall, we identified nine major immune cell categories: B cells, CD4 Tconv cells, CD8+ T cells, CD8 Tex cells, DCs, ILCs, mast cells, monocytes/macrophages (mono/macro), NK cells, plasma cells, Tprolif cells, and Tregs (**Figure 8A**). TKT expression was significantly upregulated in mono/macro cells (**Figure 8B**), suggesting a potential association between TKT and immune cell function. Further analysis of the interactions between mono/macro and other immune cell types revealed dynamic relationships, indicating TKT’s involvement in immune cell regulation (**Figure 8C**). ScRNA-Seq data showed that TKT expression was relatively high in mono/macro, with fluctuations at specific differentiation stages, suggesting a potential role in macrophage polarization and adaptation within the tumor microenvironment (**Figures 8D–G**).

Using the Visium spatial transcriptomics platform, we evaluated the spatial distribution of TKT within tissue microenvironments (**Supplementary Figure S2**, **Figure 8H**). Spearman correlation analysis confirmed that TKT expression was predominantly enriched in macrophages, with a strong correlation observed between the two (**Figure 8I**), consistent with the ScRNA-Seq findings. This suggests that TKT may support macrophage-driven processes such as immune regulation and inflammatory responses, particularly in the context of tumor development. The tumor microenvironment was further analyzed by defining regions as “malignant” (score of 1), “normal” (score of 0), or “mixed” (regions containing both malignant and non-malignant cells). TKT expression was highest in mixed regions, followed by malignant regions, and was lowest in normal regions (**Figure 8J**). This pattern highlights the potential role of TKT in the interaction between malignant and immune cells, facilitating tumor progression. The increased expression of TKT in these regions may reflect its role in promoting immune evasion, tumor cell survival, and adaptation within the complex tumor microenvironment. These findings imply that TKT may contribute to macrophage dynamics and the crosstalk between tumor and immune cells, highlighting its potential as a therapeutic target.

### Drug susceptibility analysis

We subsequently computed IC50 values using the “oncopredict” package in R to investigate therapeutic responses, assessing patient sensitivity to various immunotherapy and chemotherapy drugs, including those in clinical trials. Patients with low TKT expression exhibited lower IC50 values for axitinib, gefitinib, and lenalidomide, suggesting an enhanced sensitivity to these treatments. Conversely, patients with high TKT expression had lower IC50 values for bortezomib, cisplatin, cyclopamine, docetaxel, gemcitabine, and sorafenib, indicating increased drug sensitivity (**Supplementary Figure S3**).

To further explore the potential role of TKT in immunotherapy, we subsequently analyzed TKT expression in HCC samples from patients treated with nivolumab (PD-1 inhibitor) and cabozantinib (a multi-targeted tyrosine kinase inhibitor), using the GSE238264 dataset. We categorized patients into responding and non-responding groups, based on the response to immunotherapy, and compared the differences in TKT expression between the two groups. The results showed that the expression of TKT in tumor tissues of patients with poor response to immunotherapy was significantly higher than that of patients with a good response (**Supplementary Figure S4**). Further analysis revealed that TKT expression was generally higher in non-responding patients than in responding patients in various immune cells. However, the results were reversed in macrophages, for which responding patients had significantly higher levels of TKT expression. This finding suggests that TKT expression may play an important role in the efficacy of immunotherapy by regulating the metabolism and function of immune cells and thereby influencing the characteristics of the tumor microenvironment.

### The oncogenic effect of TKT is dependent on reprogrammed glucose metabolism in Hep-G2 cells

The Warburg effect, which promotes cancer cell growth and resistance to therapeutic interventions, is characterized by glucose metabolism reprogramming. TKT, a critical enzyme linking the PPP and glycolysis, plays a central role in this metabolic shift, suggesting its potential influence on cancer cell metabolism. We therefore hypothesized that elevated TKT expression in cancer facilitates tumor growth and metastasis by altering glucose metabolism.

**Figure 9:**
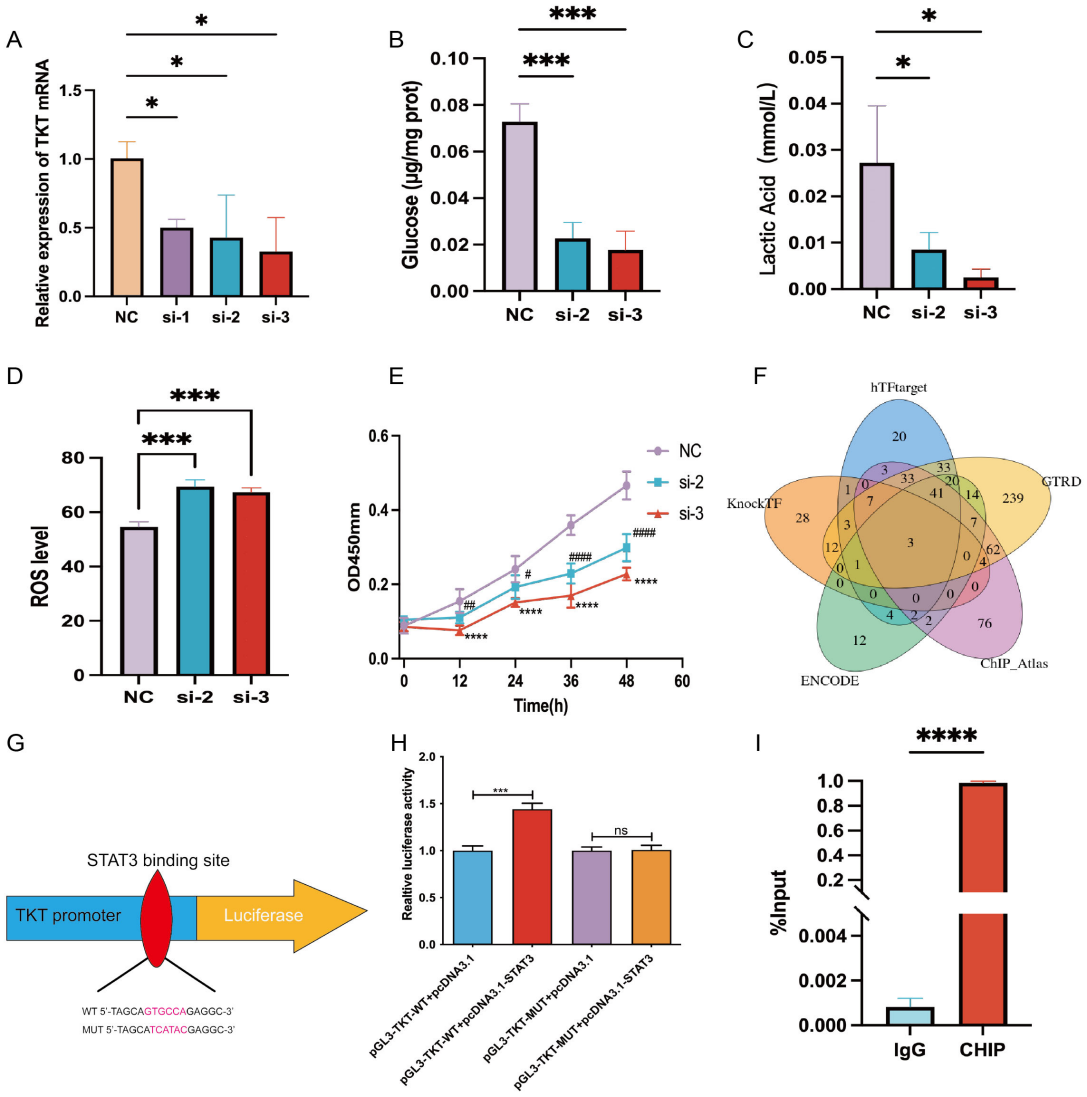
Experimental verification of TKT. **(A)** TKT mRNA levels were assessed in Hep-G2 cells, negative control (NC), and siRNA-treated subclones (si-1, si-2, si-3), with NC serving as the internal control. **(B)** Glucose uptake assay in NC, si-2, and si-3 subclones. **(C)** Lactic acid assay in NC, si-2, and si-3 subclones. **(D)** Intracellular ROS levels were measured following TKT knockdown. **(E)** Cell proliferation assay in NC, si-2, and si-3 subclones. **(F)** Prediction of TFs potentially binding to TKT using five databases. **(G)** Binding sequence of STAT3 to TKT predicted from the JASPAR database. **(H)** Verification of the transfectant’s impact on STAT3 interaction with the TKT promoter through a luciferase reporter assay in HCC cells. **(I)** ChIP assay results confirm direct binding between STAT3 and the TKT promoter region. (*,#P <0.05, **,##P < 0.01, ***,###P < 0.001, ****,####P < 0.0001, and ns means no significance).

TKT knockdown (**Figure 9A**) significantly reduced glycolytic activity in Hep-G2 cells, as demonstrated by decreased glucose uptake and lactic acid production (**Figures 9B, C**). Additionally, TKT depletion resulted in increased ROS levels (**Figure 9D**), indicating its role in maintaining redox homeostasis. TKT knockdown also inhibited Hep-G2 cell proliferation (**Figure 9E**), highlighting its role in driving oncogenic processes. These findings suggest that TKT promotes cancer cell growth and survival by reprogramming glucose metabolism, making it a potential target for therapeutic strategies aimed at inhibiting cancer cell metabolism and proliferation.

### STAT3 regulation of TKT

Using five TF prediction tools and correlational studies of TKT expression, we identified three genes that consistently correlated with TKT expression: MYC, GATA2, and STAT3 (**Figure 9F**). Among these, we focused on STAT3, a key regulator of glucose and lactic acid metabolism in HCC (22, 23). To substantiate STAT3’s role in the transcriptional regulation of TKT, we engineered wild-type (WT) and mutant (MUT) TKT plasmids based on the predicted STAT3 binding site (GTCCTG) identified through JASPAR promoter analysis (**Figure 9G**). Dual-luciferase assays confirmed STAT3 binding to this site (**Figure 9H**). Further validation using ChIP-qPCR analysis demonstrated direct STAT3 binding to the TKT promoter region (**Figure 9I**), highlighting STAT3 as a critical regulator of TKT expression. Given STAT3’s established role in modulating glucose metabolism, this regulation suggests a direct link between STAT3 and TKT in cancer cell metabolism reprogramming.

These findings reveal a dual regulatory mechanism: STAT3-mediated transcriptional activation promotes TKT expression, while hypomethylation of the promoter region further enhances the transcriptional activity of TKT. Activation of TKT by STAT3 may drive metabolic reprogramming by enhancing the flow of glucose in the PPP, thereby enhancing cancer cell survival and proliferation. Hypomethylation of the promoter region promotes the binding of TKT to TFs, further facilitating its expression and function. This complex interplay of epigenetic and transcriptional regulation highlights the central role of TKT in cancer metabolism, suggesting its potential as a therapeutic target, particularly in terms of inhibiting the STAT3-TKT signaling pathway and regulating TKT expression.

## Discussion

The role of TKT in different cancers demonstrates its dual role as both a potential risk factor and a protective factor, a phenomenon that highlights the complexity of its function. As a key enzyme of the PPP, TKT plays an important role in supporting rapid cell proliferation and growth; however, under certain conditions it may also exert a protective role. This dual function of TKT may be closely related to the metabolic requirements of different tumor types, as well as to the characteristics of the TME. Overexpression of TKT in tumor types with faster proliferation rates (e.g., pancreatic and HCC) is usually associated with a poor prognosis, because TKT supports tumor cell growth and survival by enhancing the activity of the PPP, promoting nucleotide synthesis and enhancing cellular antioxidant capacity. In such tumors, high expression of TKT helps tumor cells to better cope with metabolic stress and oxidative damage, which in turn increases their drug resistance and enhances metastasis. In contrast, in less metabolically active tumor types (e.g., UVM), TKT may play a protective role by maintaining redox homeostasis. Oxidative stress, particularly ROS accumulation, plays a key role in tumorigenesis and the pathology of certain ocular diseases (24–26). In these tumors, TKT expression reduces oxidative damage, thereby protecting tumor cells from the adverse impacts of metabolic imbalance. This protective effect may explain the association between TKT in UVM and better prognosis. Thus, the dual role of TKT in different cancers reflects its key role in the complex interaction between metabolic demands and the tumor microenvironment.

HCC is known for its highly malignant and aggressive nature, which contributes to unfavorable outcomes, primarily due to drug resistance, recurrence, and metastasis (27–29). HCC exhibits significant heterogeneity, with various molecular subtypes coexisting within individual tumor lesions (30). These subtypes exhibit unique biological properties and marked differences in key malignant behaviors such as proliferation, invasion, and drug resistance, all of which are essential for tumor progression (31). This study found high TKT levels in clinical samples of HCC, Hep-G2 cell lines, and datasets from the TCGA and GEO databases. Additionally, elevated TKT levels were identified in mono/macro from patients with HCC in the GSE140228 dataset. Previous studies have established a connection between dysregulated TKT expression and metabolic reorganization, a foundational mechanism driving malignant progression (6, 32, 33). Prior studies have shown that TKT is associated with a poorer prognosis in colorectal cancer, due to its promotion of metastasis (34, 35). Similarly, in our analysis of the GSE97626 dataset, we found that HCC cell lines MHCC97L and HCCLM3 with high metastatic potential had significantly higher TKT expression levels than Huh7 cells with low metastatic potential. This indicates that TKT may play a crucial role in the metastatic process of HCC. Overall, the findings of the present study align with those of Li et al. (36).

Thanks to the rapid development of tumor immunotherapy and microarray sequencing technology, bioinformatics has played an increasingly important role in the exploration of new targets for HCC immunotherapy. Studies have shown that the difference between immune score and stromal score significantly affects the survival and prognosis of patients with liver cancer (37). In addition, higher immune scores in tissues adjacent to HCC were found to be associated with earlier recurrence, further confirming the critical role of immune cells in the development and progression of HCC (38). Therefore, the ESTIMATE score, as an effective tool to comprehensively evaluate the content of immune cells and stromal cells in tissues, has significant potential and is expected to become an important indicator to predict patient prognosis.

Tumor cells drive tumor progression by interacting with and adapting to their microenvironment. A growing array of promising immunotherapies is currently under clinical investigation for cancer treatment. This study noted that TKT was positively correlated with eosinophils, macrophages M0, and Tregs in HCC tissues. Tregs are crucial in curbing autoimmunity and provide effective anti-tumor immunity. Increased Treg infiltration within tumor sites is frequently correlated with worse outcomes in patients with cancer (39). Moreover, TKT is significantly associated with various ICPs and immunomodulatory factors. Given their critical roles in immune evasion, ICPs have become focal points in pharmaceutical investigations, and their blockade represents the most promising strategy for cancer immunotherapy (40). Our analysis of HCC using the TCGA database confirmed the correlation of TKT with additional ICP genes, including CD44, CD80, CD86, and CTLA-4, which may explain the association of TKT overexpression with poorer prognoses in patients with cancer. In HCC, TKT expression positively correlates with tumor stemness, which promotes tumor initiation, metastasis, and drug resistance. Common mutations in patients with HCC, such as TP53 and CTNNB1, may influence the effectiveness of ICP inhibitor (ICI) therapy (41). TP53 mutations can drive immune evasion in HCC cells, potentially diminishing the effectiveness of ICI therapy. Additionally, alterations in CTNNB1 and AXIN1 may modify the Wnt/β-catenin pathway (42), thus affecting the responsiveness of HCC cells to ICI therapy. Although there are currently no studies directly exploring the relationship between TKT and TMB, its biological role indicates that high TKT expression could indirectly contribute to elevated TMB by increasing genomic instability in tumor cells. This, in turn, may influence the response to immunotherapy. While TMB serves as an effective predictive biomarker for immunotherapy across various cancers, its limitations as a standalone marker are evident. Consequently, combining TKT expression with TMB analysis could offer a more comprehensive and reliable basis for the prediction of responses to personalized immunotherapy. Although further research is needed to explore the relationship between TKT and TMB, as well as to assess their clinical relevance across different tumor types, this approach could ultimately lead to the development of more accurate biomarker combinations for optimizing immunotherapy strategies. These findings suggest a correlation between TKT and tumor immunity, highlighting TKT’s role in tumor development and its potential impact on the effectiveness of immunotherapy.

In recent years, significant progress has been made in the systematic treatment of HCC, particularly in the fields of targeted therapy, immunotherapy and chemotherapy combination therapy. As the first systemic treatment approved by the FDA, Sorafenib has demonstrated significant efficacy in HCC patients, and has arisen as a cornerstone of clinical treatment. However, there are significant differences in how individual patients respond to the drug. In our study, it was found that groups with high TKT expression were more sensitive to sorafenib and cisplatin. We further found that low TKT expression may be associated with better immunotherapy responses. This finding provides a new perspective regarding the role of TKT in tumor metabolism. Specifically, that TKT may regulate the response of HCC to different drug therapies through metabolic reprogramming in tumor cells, thereby affecting its efficacy. As such, therapeutic strategies targeting TKT may enhance the sensitivity of tumor cells to existing targeted therapies and immunotherapies by influencing metabolic homeostasis, thereby optimizing the comprehensive treatment regimen for HCC.

TKT inhibitors, such as oxygen thiamine (OT) and Oroxylin A, are gaining increasing attention for their ability to disrupt key metabolic processes that support cancer cell proliferation and therapeutic resistance. OT inhibits TKT, which requires ThDP (thiamine pyrophosphate), the active form of thiamine, as a cofactor for its activation (43–46). By interfering with nucleic acid synthesis and redox balance, OT modulates the PPP, thereby disrupting essential metabolic processes in cancer cells. This not only suppresses tumor growth, but also increases ROS levels, increasing the sensitivity of cancer cells to chemotherapy. Combining OT with chemotherapeutics like sorafenib, imatinib, cisplatin, or gemcitabine has shown promise in restoring drug sensitivity in resistant cancers, such as chronic myelogenous leukemia, and pancreatic cancer (47). Additionally, OT has demonstrated synergy with docetaxel and doxorubicin in the treatment of triple-negative breast cancer (48). In the future, combining TKT inhibitors like OT and Oroxylin A with traditional chemotherapy holds significant potential as a strategy to overcome resistance and enhance treatment outcomes across a wide range of cancers.

Similarly, Oroxylin A, a selective TKT inhibitor, has shown strong preclinical evidence in inhibiting HCC growth. By targeting the non-oxidative branch of the PPP, Oroxylin A disrupts nucleotide synthesis and redox homeostasis, both of which are essential for cancer cell metabolism and survival. Studies in mouse models and patient-derived organoids have shown that Oroxylin A significantly reduces HCC tumor growth. These results indicate that targeting the non-oxidative PPP could be a novel strategy for treating HCC and potentially other cancers (49). Combining TKT inhibitors like OT and Oroxylin A with traditional chemotherapy may also offer a promising approach to overcome resistance and improve treatment outcomes across various cancers. It is important to note that TKT has a dual function. Although overexpression of TKT is one of the key factors in the malignant progression of hepatocellular carcinoma, its role in normal growth and development is equally crucial. Indeed, studies have shown that TKT deficiency disrupts the normal function of the intestinal barrier, which in turn leads to enteritis. In addition, decreased TKT activity has been implicated as a driving factor in the development of diabetic complications (50). By activating TKT, not only can diabetic retinopathy and cardiomyopathy be prevented, but the healing of limbs in diabetic patients can also be promoted, while the symptoms of diabetic nephropathy can be alleviated (51). More critically, TKT inhibitors may trigger side effects when used in combination with chemotherapeutic agents. For example, TKT inhibitors counteract each other’s antitumor effects when used in combination with lovastatin (52). Therefore, more detailed safety and efficacy evaluations must be performed before TKT inhibitors can be used in clinical application.

This study has certain limitations. First, some findings were based on a single approach or database, without cross-verification from multiple sources. Second, although our bioinformatics analyses indicated an association between TKT and unfavorable outcomes in HCC and immune responses, it remains uncertain whether TKT affects prognosis by regulating immune processes. Finally, while our findings suggest promising directions for future studies, additional experimental validation is essential to elucidate the specific biological functions and molecular mechanisms involved. In summary, through this multi-omics approach and experimental analysis, we provide a holistic view of TKT function in immunotherapy and prognosis, emphasizing its role in HCC. The insights gained from this study may inform future therapeutic strategies targeting TKT and enhance our understanding of its impact on cancer progression and treatment responses.

## Data Availability

The original contributions presented in the study are included in the article/**Supplementary Material**. Further inquiries can be directed to the corresponding author.
