# Supplementary material for: The role of transketolase in the immunotherapy and prognosis of hepatocellular carcinoma: a multi-omics approach
Source: Front Immunol. 2025 Mar 31;16:1529029. doi: 10.3389/fimmu.2025.1529029 (PMC11994433; doi:10.3389/fimmu.2025.1529029)
Supplement: Supplementary file 1 [file DataSheet1.docx]

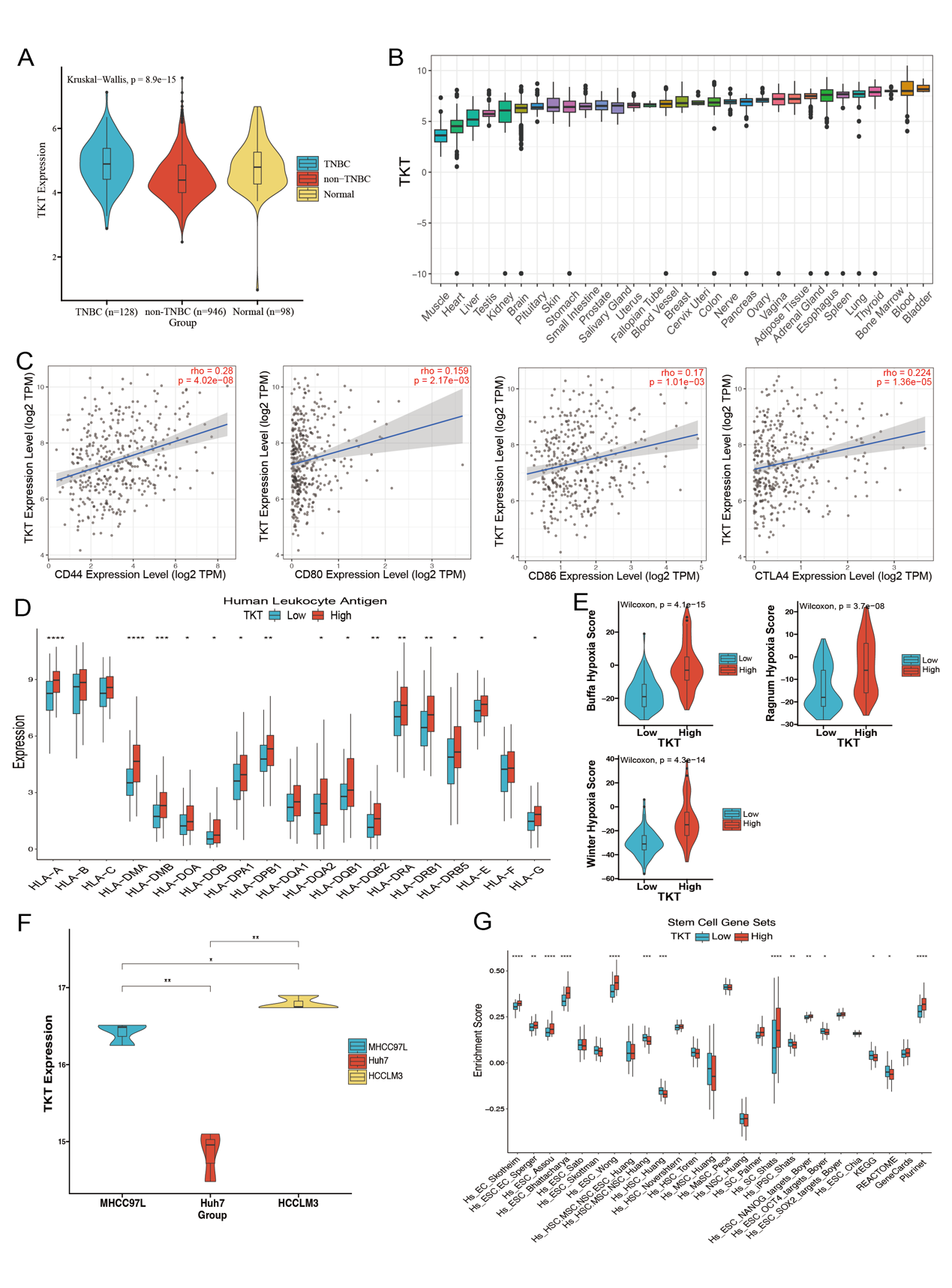


Figure S1. Analysis of TKT expression across various conditions and its correlation with tumor markers, hypoxia scores, and stem cell gene sets (A) Comparison of TKT expression in TNBC, non-TNBC, and normal samples. (B) TKT expression across various human tissues from GTEx dataset. (C) Analysis of the correlation between TKT and CD44, CD80, CD86, and CTLA-4. (D) Correlation analysis of 19 HLA-related genes with TKT expression. (*P < 0.05, P < 0.01, P < 0.001) (E) Calculation of hypoxia scores using three algorithms and their correlations with TKT expression. (F) TKT expression in different HCC cell lines. (G) Correlation analysis of TKT with stemness genes in 26 datasets.

Figure S2. Spatial distribution heatmap of different cell types in hepatocellular carcinoma tissues.


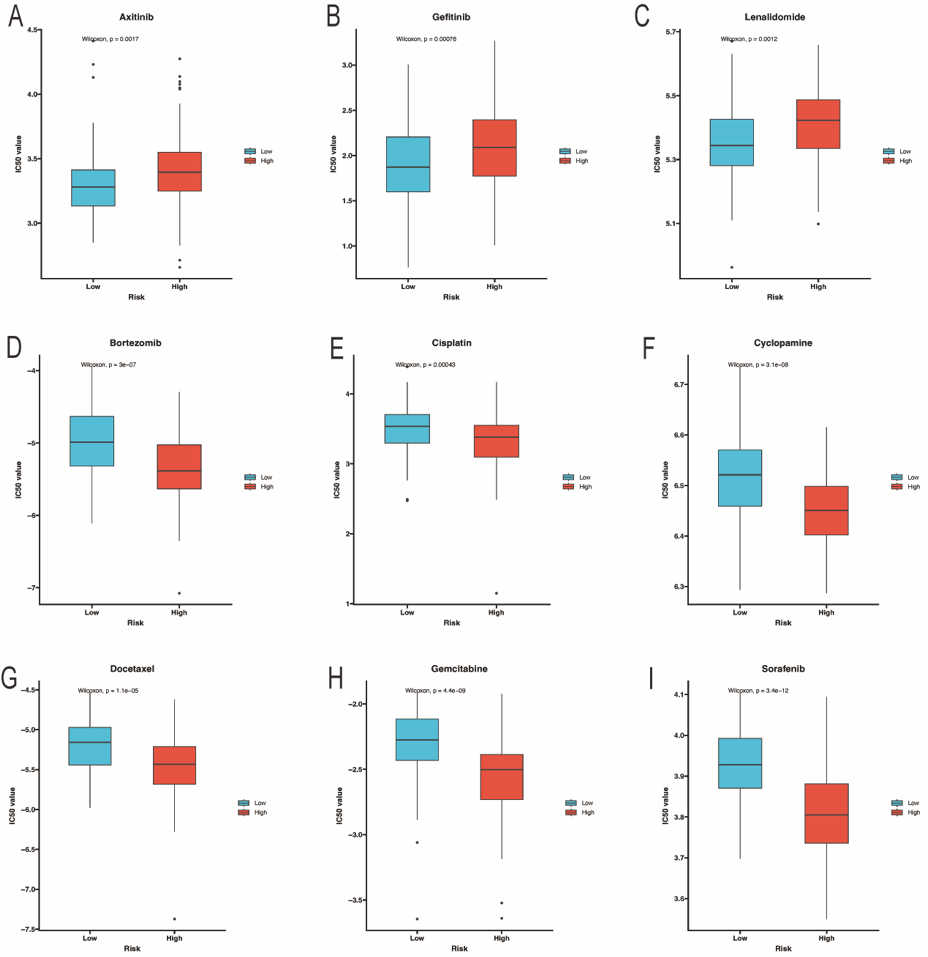


Figure S3. Drugs sensitivity analysis of TKT. (A)Axitinib, (B) Gefitinib, (C) Lenalidomide, (D)Bortezomib, (E) Cisplatin, (F) Cyclopamine, (G) Docetaxel, (H) Gemcitabine and (I) Sorafenib.


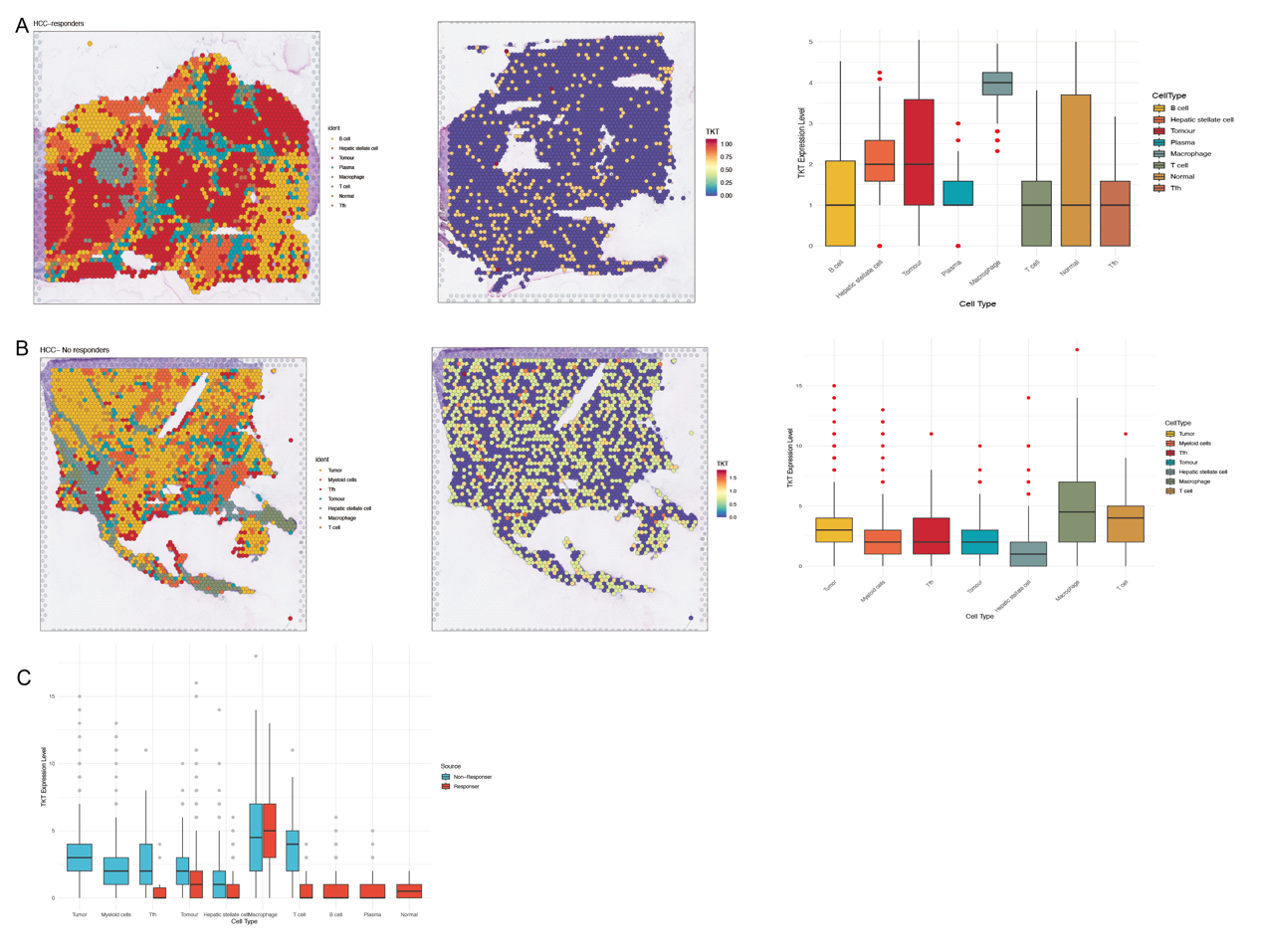


Figure S4. Analysis of TKT expression and cell type distribution in HCC responders and non-responders. (A) TKT expression and cell type distribution in HCC responders. (B) TKT expression and cell type distribution in HCC non-responders. (C) Comparison of TKT expression in different cell types between responders and non-responders.
