# Supplementary material for: The role of transketolase in the immunotherapy and prognosis of hepatocellular carcinoma: a multi-omics approach
Source: Front Immunol. 2025 Mar 31;16:1529029. doi: 10.3389/fimmu.2025.1529029 (PMC11994433; doi:10.3389/fimmu.2025.1529029)
Supplement: Supplementary file 2 [file Table1.docx]

Supplemental Table 1. qPRC primers of *TKT and* β-actin

| Gene | Sense (5’-3’) | Antisense (5’-3’) |
| --- | --- | --- |
| *TKT* | CCTACACCGGCAAATACTTCG | GCCTCCCATACAGAGCCCT |
| β-actin | GTCATTCCAAATATGAGATGCGT | GCTATCACCTCCCCTGTGTG |
| *TKT-CHIP* | CACACAGCTTGTGAAGAAGA | ATTATACTCTAAGTCTTCTC |
