# Supplementary material for: The role of transketolase in the immunotherapy and prognosis of hepatocellular carcinoma: a multi-omics approach
Source: Front Immunol. 2025 Mar 31;16:1529029. doi: 10.3389/fimmu.2025.1529029 (PMC11994433; doi:10.3389/fimmu.2025.1529029)
Supplement: Supplementary file 3 [file Table2.docx]

Supplemental Table 2. siRNA of *TKT*

| Gene | Sense (5’-3’) | Antisense (5’-3’) |
| --- | --- | --- |
| *Si-1* | GCUCAAAGAUGUACUGAGA | UCUCAGUACAUCUUUGAGC |
| Si-2 | GGCUGUGUCCAGUGCAGUA | UACUGCACUGGACACAGCC |
| *Si-3* | GCUACAUUGCUGAGCAGAA | UUCUGCUCAGCAAUGUAGC |
